# Broad innate immune activation enhances the protective efficacy of rBCG-LTAK63 against *Mycobacterium tuberculosis*

**DOI:** 10.3389/fimmu.2026.1758476

**Published:** 2026-03-04

**Authors:** Ana Carolina de Oliveira Carvalho, Monalisa Martins Trentini, Dunia Rodriguez, Lázaro Moreira Marques-Neto, Paulo Henrique Santana Silveira, Nancy Starobinas, Sergio Costa Oliveira, Luciana Cezar de Cerqueira Leite, Alex Issamu Kanno

**Affiliations:** 1 Laboratório de Desenvolvimento de Vacinas, Instituto Butantan, São Paulo, Brazil; 2 Laboratório de Imunogenética, Instituto Butantan, São Paulo, Brazil; 3 Departamento de Imunologia, Instituto de Ciências Biomédicas, Universidade de São Paulo, São Paulo, Brazil

**Keywords:** BCG vaccine, inflammasome, innate immunity, rBCG-LTAK63, recombinant BCG, tuberculosis

## Abstract

**Introduction:**

Tuberculosis (TB) continues to be one of the leading infectious causes of mortality world-wide, while the Bacillus Calmette–Guérin (BCG) vaccine provides variable protection. To address this limitation, our group developed a recombinant BCG strain expressing a detoxified *Escherichia coli* heat-labile toxin subunit (rBCG-LTAK63), which confers superior protection against *Mycobacterium tuberculosis* (Mtb). However, the mechanisms underlying this enhanced efficacy remain to be better characterized. Here, we investigated the capacity of rBCG-LTAK63 to enhance inflammasome-associated innate immune responses and its impact on T cell activation and protection against Mtb.

**Methods:**

Bone marrow-derived macrophages (BMDMs) from wild-type (C57Bl/6), *Casp-1*-/-, *Nlrp3*-/-, *Aim-2*-/- and phenotypically selected AIRmax and AIRmin^TT^ mice were used to evaluate inflammasome-associated responses using IL-1β as a primary readout. A co-culture system of inflammasome-activated BMDMs and splenocytes was employed to assess CD4^+^ T cell activation and polarization. Additionally, immunization of AIRmax and AIRmin^TT^ mice with BCG or rBCG-LTAK63 was performed to evaluate protection against Mtb.

**Results:**

rBCG-LTAK63 induced significantly higher IL-1β production than parental BCG. IL-1β production was largely ASC-associated and predominantly dependent on the NLRP3/caspase-1 axis; however, residual IL-1β production was still detected in *Casp*-/- and *Nlrp3*-/- BMDMs, indicating the contribution of additional processing pathways. Co-culture of inflammasome-activated BMDMs and splenocytes showed that rBCG-LTAK63-primed macrophages strongly promoted CD4^+^ T cell activation and polarization toward Th1/Th17 responses. *In vivo*, BCG only induced protection in AIRmax mice, while rBCG-LTAK63 induced protection in both AIRmax and AIRmin^TT^ genotypes.

**Conclusion:**

This demonstrates that protection is achieved in a diminished innate inflammatory response scenario, but its full engagement can further reduce pulmonary bacterial loads. Together, these findings demonstrate that rBCG-LTAK63 enhances protection against TB through a broad innate activation including inflammasome-associated mechanisms.

## 1 Introduction

Tuberculosis (TB) remains the leading cause of death from a single infectious agent worldwide. In 2023, according to the World Health Organization (WHO), TB was responsible for approximately 1.5 million deaths. The Bacillus Calmette–Guérin (BCG) has been the only WHO-licensed vaccine against TB for over a century (1). However, several studies have shown that BCG confers variable protection in adults. Consequently, there is an urgent need to develop more effective vaccines (2).

Successful vaccines engage innate immune pathways through the use of adjuvants to promote the development of an effective adaptive immunity. Inflammasomes are cytosolic multiprotein complexes that play a critical role in innate immunity by detecting danger signals and initiating inflammatory responses (3). Their activation is initiated by pattern recognition receptors (PRRs) that detect pathogen- (PAMPs) or damage-associated molecular patterns (DAMPs). Once activated, inflammasomes trigger the cleavage of pro-caspase-1 into its active form, leading to the maturation and release of IL-1β and IL-18, which orchestrate the early immune response against invading pathogens (4).

IL-1β is a central proinflammatory cytokine. It enhances dendritic cell activation and co-stimulation, boosts antigen presentation and cross-priming, recruits and activates neutrophils, and, together with IL-6/IL-23/TGF-β, drives RORγt in naïve T cells guiding them to Th17 differentiation. All together innate-derived cytokines, costimulatory signals, and the dose/duration of antigen presentation guide the quality of the response (e.g., Th1/Th2/Th17 polarization) (5).

Recent studies have highlighted the relevance of inflammasome activation in host defense against mycobacterial infections and in the protective efficacy of TB vaccines (6–11). One promising approach in TB vaccine development is the use of recombinant BCG strains expressing adjuvant molecules that can enhance the innate immune response and improve adaptive immune response (12, 13). Our group developed a recombinant BCG strain, rBCG-LTAK63 (rBCGΔlysA::lysA-ltak63), that uses auxotrophic complementation to stably express the genetically detoxified A subunit of Escherichia coli heat-labile toxin (14). Previous studies demonstrated that immunization with rBCG-LTAK63 provided enhanced protection against *Mycobacterium tuberculosis* (Mtb) H37Rv challenge compared to the parental BCG strain (15), likely due to the induction of a stronger inflammatory response (16). More recently, we showed that rBCG-LTAK63 elicits a Th1/Th17 adaptive immune response, increased memory T cell populations and long-term protection (up to 180 days after immunization) (17). Moreover, innate immune responses induced by rBCG-LTAK63 are also distinct from those generated in response to wild-type BCG. Increased numbers of macrophages and neutrophils are seen after the intraperitoneal immunization of mice with rBCG-LTAK63 (18). Stimulation of human macrophages with rBCG-LTAK63 resulted in an increased inflammatory response (19). However, the mechanisms through which LTAK63 expression in BCG augments the Th1/Th17 response are not completely understood.

Recombinant forms of the *E. coli* heat-labile enterotoxin are reported to activate antigen-presenting cells (APCs) and upregulate surface markers such as MHC-II, CD40, CD86, and promote the production of IL-1β (20). Based on these observations, we hypothesized that rBCG-LTAK63 enhances innate immune signaling, including inflammasome-associated pathways, thereby contributing to improved vaccine efficacy. In the present study, we investigated the capacity of rBCG-LTAK63 to induce inflammasome-associated responses and evaluated how these innate mechanisms relate to T cell activation and protection against *M. tuberculosis*.

## 2 Materials and methods

### 2.1 Ethical statement and mice

All animal procedures were approved by the Ethics Committee on Animal Use of the Butantan Institute (CEUA/IB protocol 2012250722) and conducted in compliance with the Brazilian National Council for Animal Experimentation Control (CONCEA) guidelines. Female C57Bl/6 mice (4–8 weeks old) were obtained from the Butantan Institute’s Central Animal Facility. Knockout strains, *Casp-1*-/-, *Nlpr3*-/-, and *Aim-2*-/- (C57Bl/6 background) were kindly provided by Dr. Sérgio Costa Oliveira (University of São Paulo). AIRmax and AIRmin^TT^ mice are bred under standardized conditions at the Laboratório de Imunogenética (21). Mice were housed in the Laboratório de Desenvolvimento de Vacinas facility with ad libitum access to food and water. Environmental conditions were maintained at 20–24 °C, 40–70% relative humidity, and a 12-h light/dark cycle.

### 2.2 Bacterial strains and culture conditions

The recombinant rBCG-LTAK63 strain (ΔlysA::lysA-ltak63), generated from BCG Danish as previously described (14) and control strain (BCG Danish ATCC 35733, American Type Culture Collection, Rockville, MD, USA) were cultured in Middlebrook 7H9 (BD Difco™, Detroit, MI, USA) medium supplemented with 10% OADC (oleic acid–albumin–dextrose–catalase; BD BBL, Cockeysville, MD, USA), 0.05% Tween 80 (Sigma-Aldrich^®^, Merck KGaA, St. Louis, MO, USA), and 0.5% glycerol (Sigma-Aldrich) at 37 °C with 5% CO_2_ for 7 days. Cultures were expanded to 50 mL and grown to mid-log phase (OD_600_ = 0.8). Bacterial cells were harvested by centrifugation at 3,180 × g, washed twice with ice-cold 10% glycerol, and resuspended in 1 mL of the same solution. Aliquots (100 µL) were cryopreserved at -80 °C for long-term storage. Post-preservation viability was determined by colony-forming unit (CFU) counts. Serial dilutions were plated on Middlebrook 7H10 (BD Difco™) agar supplemented with 10% OADC (BD BBL™) and incubated for 21 days at 37 °C with 5% CO_2_.

### 2.3 RNA-seq

The dataset is deposited in GEO under accession GSE278523, and the corresponding raw sequencing data are available in NCBI SRA under BioProject PRJNA1159865. BALB/c mice were immunized with rBCG-LTAK63 or BCG (10^6^ CFU, s.c.) and lymph node samples collected at 7 days post-immunization. Gene expression was analyzed using EdgeR (22). Raw gene counts were normalized using the trimmed mean of M-values (TMM) method to correct for library size variations. Differentially expressed genes (DEGs) were identified using a false discovery rate (FDR) threshold of < 0.05, with multiple testing correction applied via the Benjamini-Hochberg method. To investigate functional relationships among DEGs, protein-protein interaction (PPI) networks were constructed using the Search Tool for the Retrieval of Interacting Genes/Proteins (STRING) database v11.5, applying a medium confidence interaction score (≥ 0.4). Subsequently, KEGG pathway enrichment analysis was conducted to identify significantly overrepresented biological pathways within the DEG dataset, with statistical significance defined as FDR < 0.05.

### 2.4 Isolation and differentiation of murine bone marrow macrophages

C57Bl/6, *Casp-1*-/-, *Nlrp3*-/-, *Aim-2*-/-, AIRmax, and AIRmin^TT^ mice were euthanized with an overdose of anesthetic (300 mg/kg ketamine + 30 mg/kg xylazine) by intraperitoneal route, and tibias and femurs were aseptically collected. Bone marrow cells were flushed from the bones using RPMI-1640 medium (Gibco, Life Technologies, Paisley, UK) and a 10 mL disposable syringe (BD Plastipak™) with a 25G needle (BD PrecisionGlide™). The resulting cell suspension was centrifuged at 200 × g for 10 min at 4 °C. The pellet was resuspended in complete R10 medium (RPMI-1640 supplemented with 10% heat-inactivated fetal bovine serum (FBS; Sigma-Aldrich^®^)), an antibiotic solution (penicillin 100 U/mL, streptomycin 100 µg/mL), and 30% L929-conditioned medium as a source of macrophage colony-stimulating factor (M-CSF). L929 cells were originally obtained from ATCC. Cells were seeded into 6-well plates (Corning^®^, NY, USA) and incubated at 37 °C with 5% CO_2_ for 6 days, with half of the medium replaced on day 4. On day 6, non-adherent cells were removed by washing twice with phosphate-buffered saline (PBS, 137 mM NaCl, 2.7 mM KCl, 8 mM Na2HPO4, and 1.5 mM KH2PO4, pH 7.2), and adherent cells were detached using a cell scraper (KASVI, Pinhais, PR, Brazil) in ice-cold RPMI-1640. Cells were centrifuged at 200 × g for 10 min and resuspended in complete R10 medium.

Cell viability was assessed by Trypan Blue (Sigma-Aldrich^®^) exclusion, and viable cells were counted using a Neubauer chamber. Macrophages were adjusted to 1 × 10^6^ cells/mL and seeded into 96-well plates (100 µL/well) for subsequent experiments. An aliquot was stained to confirm differentiation to BMDM by flow cytometry using anti-mouse CD11b conjugated to PE (BD Biosciences, San Diego, CA, USA) and anti-mouse F4/80 conjugated to Bv421 (BioLegend, San Diego, CA, USA).

### 2.5 Inflammasome activation assay

BMDMs (1 × 10^5^ cells/well) were plated in 96-well plates (Corning^®^) and either primed with LPS (500 ng/mL, Sigma-Aldrich^®^) for 4 h or left unstimulated, followed by infection with BCG or rBCG-LTAK63 strains at a multiplicity of infection (MOI) 10:1 for 6 h. Appropriate positive controls were included for each experiment: Nigericin (20 μM, 40 min) or ATP (5 mM, 30 min; both from Sigma-Aldrich^®^) for NLRP3 inflammasome activation. Methodologically, replacing Nigericin with ATP enhanced experimental consistency. Culture supernatants were collected and stored at -80 °C until use.

### 2.6 *In vivo* immunization and *ex vivo* co-culture assay

Female C57Bl/6 mice (5–7 weeks old) were randomly divided into three groups (n=5/group) receiving either: Saline (100 µL, subcutaneous (s.c.)), BCG Danish (100 µL, 10^6^ CFU, s.c.), or rBCG-LTAK63 (100 µL, 10^6^ CFU, s.c.). Thirty days later, spleens were aseptically collected for co-culture assays. Splenocytes were isolated by mechanical dissociation (Corning, Pyrex^®^ Ten Broeck Homogenizer), erythrocytes lysed by incubation with sterile ultrapure water for 10 s, and resuspended in complete R10 medium after centrifugation (200 × g, 10 min, 4 °C). Cell viability was assessed by trypan blue exclusion (Neubauer chamber), adjusting to 1 × 10^6^ cells/mL.

For co-culture, after differentiation BMDMs were seeded in 48-well culture plates and primed with LPS (500 ng/mL, 4 h) or left unprimed, then infected with BCG or rBCG-LTAK63 (MOI 10:1, 6 h). Controls included: unstimulated BMDMs (negative) and nigericin (48 µM, 40 min). After several PBS washes, BMDMs (2 × 10^5^/well) were co-cultured with CD28-stimulated splenocytes (2 × 10^6^/well; anti-CD28 1 µg/mL, 4 h) in 48-well plates (Corning^®^). Monensin (3 µM) was added after 48 h, and the cultures were maintained for an additional 12 h prior to analysis.

### 2.7 Immunization and challenge in AIRmax and AIRmin^TT^

Female AIRmax and AIRmin^TT^ mice (5–7 weeks old) were randomly divided into three groups receiving either: Saline (100 µL, subcutaneous (s.c.)), BCG Danish (100 µL, 10^6^ CFU, s.c.), or rBCG-LTAK63 (100 µL, 10^6^ CFU, s.c.). Thirty days later, aliquots of Mtb (H37Rv) maintained at –80 °C were thawed, and the concentration adjusted to 1.25 x 10^4^ CFU/mL using saline. Mice under mild anesthesia were instilled with 40 µL (500 CFU) into the nostrils with aid of a micropipette. Thirty days after challenge, mice were euthanized and lungs collected. For CFU counts, the cranial and median lobes of the lungs were homogenized, diluted in PBS and plated on Middlebrook 7H10 agar (BD Difco™), supplemented with 0.5% glycerol (Sigma-Aldrich^®^), 10% OADC (BD BBL™), 5 µg/ml of TCH (2-thiophenecarboxylic acid hydrazide, Sigma-Aldrich^®^, a BCG growth inhibitor) and incubated at 37 °C and 5% CO2 for up to 3 weeks to evaluate the bacterial load.

### 2.8 Quantification cytokine production

Samples were thawed and assayed for IL-1β by ELISA using the DuoSet^®^ Mouse IL-1β/IL-1F2 (R&D Systems, Inc., Minneapolis, MN, USA). For inflammation-related cytokines (IL-6, IL-10, MCP-1, IFN-γ, TNF-α, and IL-12p70) a Cytometric Beads Array (CBA mouse inflammatory kit, BD Biosciences) was used according to the manufacturer’s instructions. The assay lower limits of detection were between 2.5 and 17.5 pg/mL, depending on the cytokine, and the higher limit was 5,000 pg/mL. Data were acquired using FACS Canto II equipment and analyzed using FCAP Array Software (BD Biosciences).

### 2.9 Flow cytometry

For immunophenotyping of co-culture cells, the following extracellular markers were used: BV510-CD3 (clone: 145-2C11, BD Horizon™), FITC-CD4 (clone: GK1.5, BD Pharmingen™), PerCP-CD69 (clone: H1.2F3, BD Pharmingen™), and APC-Cy7-CD44 (clone: IM7, BioLegend). Cells were incubated with antibodies for 30 min, washed with PBS, and fixed/permeabilized using Fix/Perm Buffer (BD Biosciences™). For intracellular staining, the following markers were used: PE-IFN-γ (clone: XMG1.2, BD Pharmingen™), and BV421-IL-17 (clone: TC11-18H10, BD Horizon™). Cells were incubated with intracellular markers for 30 min, followed by washes with Perm Wash buffer (BD Biosciences™). All analyses were performed by acquiring 100,000 events on a FACSCanto II flow cytometer. Data were analyzed using FlowJo v10. Lymphocyte selection was based on size (FSC) and granularity (SSC) characteristics. CD4+ were gated to evaluate T cell activation markers (CD44+ and CD69+) and Th1/Th17-related intracellular cytokines (IFN-γ and IL-17). Gating strategy is depicted in **Supplementary Figure S1**.

### 2.10 Statistical analysis

Data was analyzed using GraphPad Prism 8.0 (GraphPad Software) and expressed as mean ± standard deviation (SD). Comparisons between two groups were performed using an unpaired two-tailed Student’s *t*-test. Multiple group comparisons were conducted using one-way analysis of variance (ANOVA), followed by Tukey’s *post hoc* test. p values < 0.05 were considered statistically significant. The experimental design figures were created with http://biorender.com.

## 3 Results

### 3.1 rBCG-LTAK63 upregulates the transcriptional signature of an early inflammatory response in mouse lymph nodes

**Figure 1:**
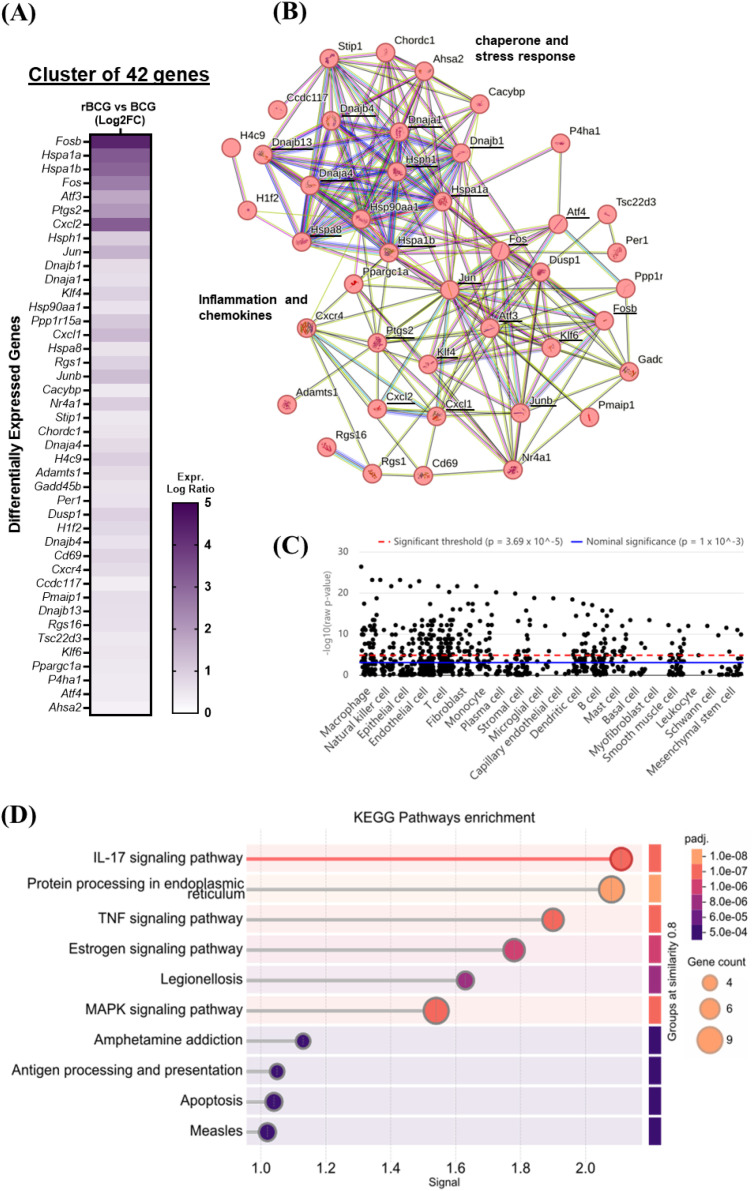
Transcriptomic analysis of the lymph nodes of mice immunized with rBCG-LTAK63. Groups of mice were immunized with BCG or rBCG-LTAK63 and the lymph nodes collected 7 days later and analyzed by RNAseq. **(A)** Heatmap and clustering of 42 differentially expressed genes (DEGs) identified in STRING (EdgeR, padj < 0.05). **(B)** Protein-protein interaction map was constructed based on the DEGs identified. Each node represents a single gene. Lines represent known/predicted interactions: light blue lines denote interactions from curated databases, representing known interactions with high confidence; pink lines indicate experimentally determined and predicted interactions; green lines represent interactions predicted by gene neighborhood analysis, red lines by gene fusion events, and dark blue lines by gene co-occurrence or other complementary methods. Additionally, yellow lines indicate interactions identified through text mining, black lines represent co-expression-based predictions, and purple lines denote interactions inferred from protein homology. **(C)** Cell-type–specific enrichment analysis of genes differentially expressed in the lymph nodes of animals immunized with rBCG-LTAK63, compared to BCG. Jitter plots display single cells from the most significantly enriched immune cell types. The dashed red line marks the Bonferroni-corrected threshold (p=3.69×10^(-5); 1,355 cell types tested), and the solid blue line marks the nominal threshold (p=1×10^(-3). **(D)** KEGG pathway enrichment analysis of DEGs. Signal represents the overall strength of the enrichment. Circle size represents the number of DEGs identified in the pathway. Statistical significance is shown by adjusted p-value (padj) (Full table as **Supplementary Table S1**).

We analyzed the transcriptomic profile of differentially expressed genes (DEGs) from the draining lymph nodes of mice at seven days post-immunization, comparing rBCG-LTAK63 to BCG using STRING analysis. A cluster of upregulated genes was revealed, indicating the transcriptional signature linked to early immune activation (Fosb, Fos, Atf3, Ptgs2), cellular stress (Hspa1a, Hspa1b, Dnajb1) and leukocyte recruitment (Cxcl1, Cxcl2) (**Figure 1A**). The protein-protein interaction map (**Figure 1B**) shows two main hubs. A chaperone-centered hub was revealed, which indicates an active and highly stressed environment, involving core (Hspa1a, Hspa1b, Hspa8), assistant co-chaperones (Dnaja4, Dnajb13, Dnajb4, Dnajb1) and other important members (Hsp90a1, Hsph1). The other hub reinforces this active immune environment linking transcription factors (Fos, Jun, Junb, Klf4, Klf6), chemokines (Cxcl1, Cxcl2), stress (Atf4, Hspa1a, Hspa1b) and inflammation resolution (Dusp1, Atf3) (**Figure 1B**).

The cell specific enrichment analysis of these genes (using human orthologues) points to expression of these genes primarily in macrophages (**Figure 1C**). The lack of direct up-regulation of IL-1 or core inflammasome genes may reflect the cellular composition of the lymph nodes, mainly T and B cells or even the time-point used (7 days after immunization). Pathway enrichment analysis (**Figure 1D**) demonstrated strong activation of key immune and inflammatory pathways, including IL-17 (mmu04657), TNF (mmu04668) and NK-κB signaling (mmu04064), antigen processing and presentation (mmu04612) and NOD-like receptor signaling (mmu04621). This molecular signature suggests a coordinated engagement of innate and adaptive immune responses, supported by an intense cellular activity demonstrated by the enrichment of Protein processing in endoplasmic reticulum (mmu04141), MAPK signaling (mmu04010) and apoptosis (mmu04210) pathways. Interestingly, the induction of pathogen-response pathways (mmu05145: Toxoplasmosis, mmu05134: Legionellosis, mmu05162: Measles) indicates broad-spectrum immune priming against intracellular pathogens.

Collectively the transcriptional analysis indicates that rBCG-LTAK63 promotes a potent and early immune response in comparison to BCG and points to macrophages as the main responsible for this response.

### 3.2 Inflammasome activation is a key innate immune mechanism triggered by the rBCG-LTAK63 vaccine

**Figure 2:**
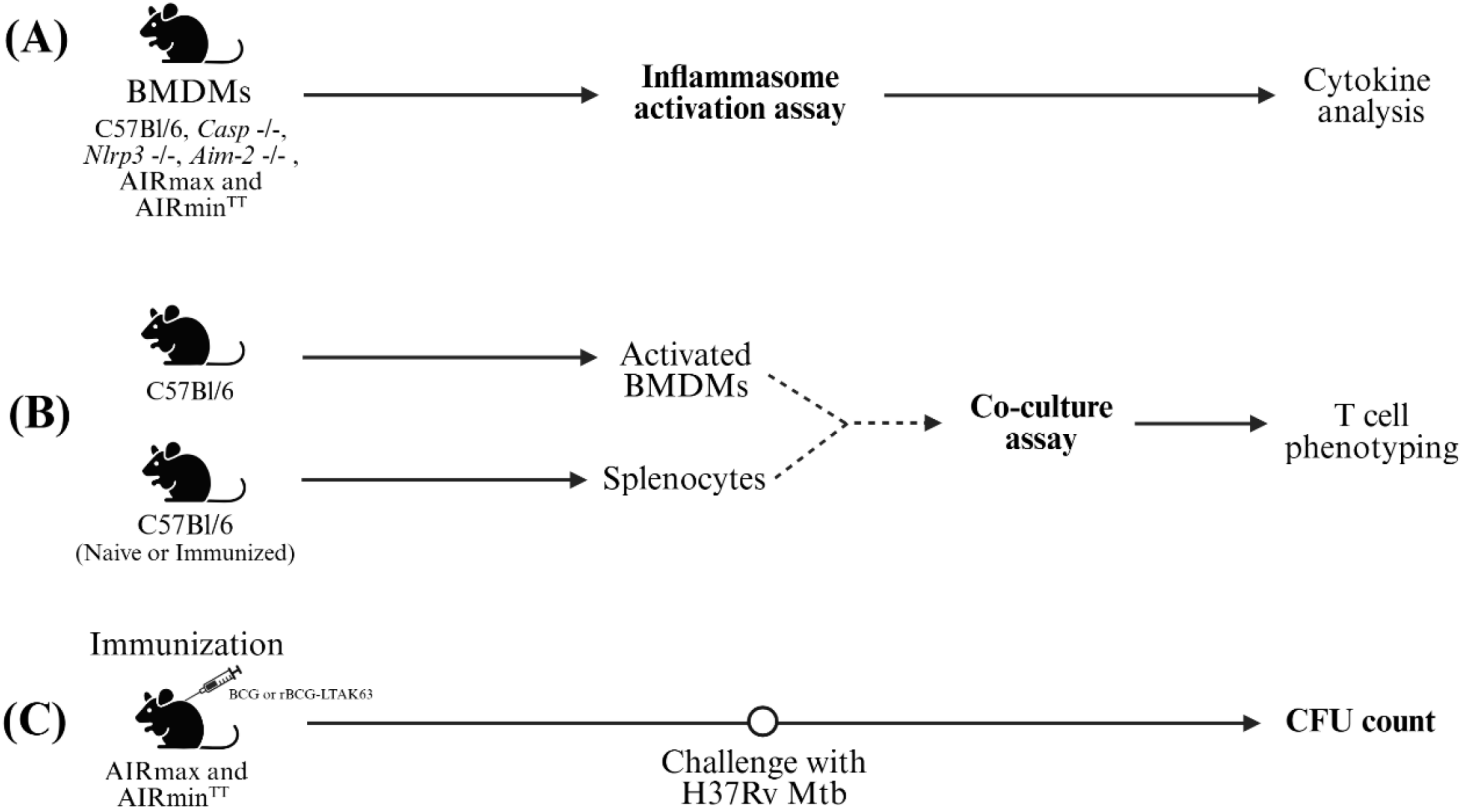
Schematic representation of the experimental design. **(A)** Inflammasome activation assay: BMDMs from C57Bl/6, *Casp-1*-/-, *Nlrp3*-/-, *Aim-2*-/-, AIRmax, and AIRmin^TT^ mice were seeded into 96-well plates (1×10^5^/well), primed (or not) with LPS (500 ng/mL, 4 h), and subsequently stimulated with BCG or rBCG-LTAK63 (MOI 10:1, 6 h). Supernatants were collected for cytokine quantification by ELISA and flow cytometry. **(B)** Co-culture assay: Spleens of naïve or immunized C57Bl/6 mice (WT) were collected thirty days after immunization. BMDMs were primed with LPS (500 ng/mL, 4 h) or left unprimed; infected with BCG or rBCG-LTAK63 (MOI 10:1, 6 h). BCG and rBCG-LTAK63 were removed by extensive washing and the cells co-cultured with splenocytes (2×10^6^/well) pre-stimulated with anti-CD28 (4 h). Activated BMDMs (2×10^5^/well) and splenocytes were cultured for 48 (h) After a monensin treatment (12 h) cells were collected and analyzed by flow cytometry. **(C)** CFU count: AIRmax and AIRmin^TT^ mice were immunized subcutaneously with 10^6^ CFU of BCG or rBCG-LTAK63. Thirty days later, mice were challenged intranasally with 500 CFU of *M. tuberculosis* H37Rv. At day 30 post-challenge, mice were euthanized, the lungs harvested, serially diluted in PBS and plated to determine CFU counts.

To characterize the contribution of inflammasome-associated pathways to the immune response induced by rBCG-LTAK63, we combined *in vitro* assays using murine macrophages with *in vivo* infection models. These analyses were performed in wild-type mice, in knockout strains lacking key inflammasome components, and in AIRmax and AIRmin^TT^ mouse lines, selected for maximal and minimal inflammatory responsiveness including inflammasome-associated responses, respectively. Bacterial burden was assessed in these models through CFU counts (**Figure 2**).

**Figure 3:**
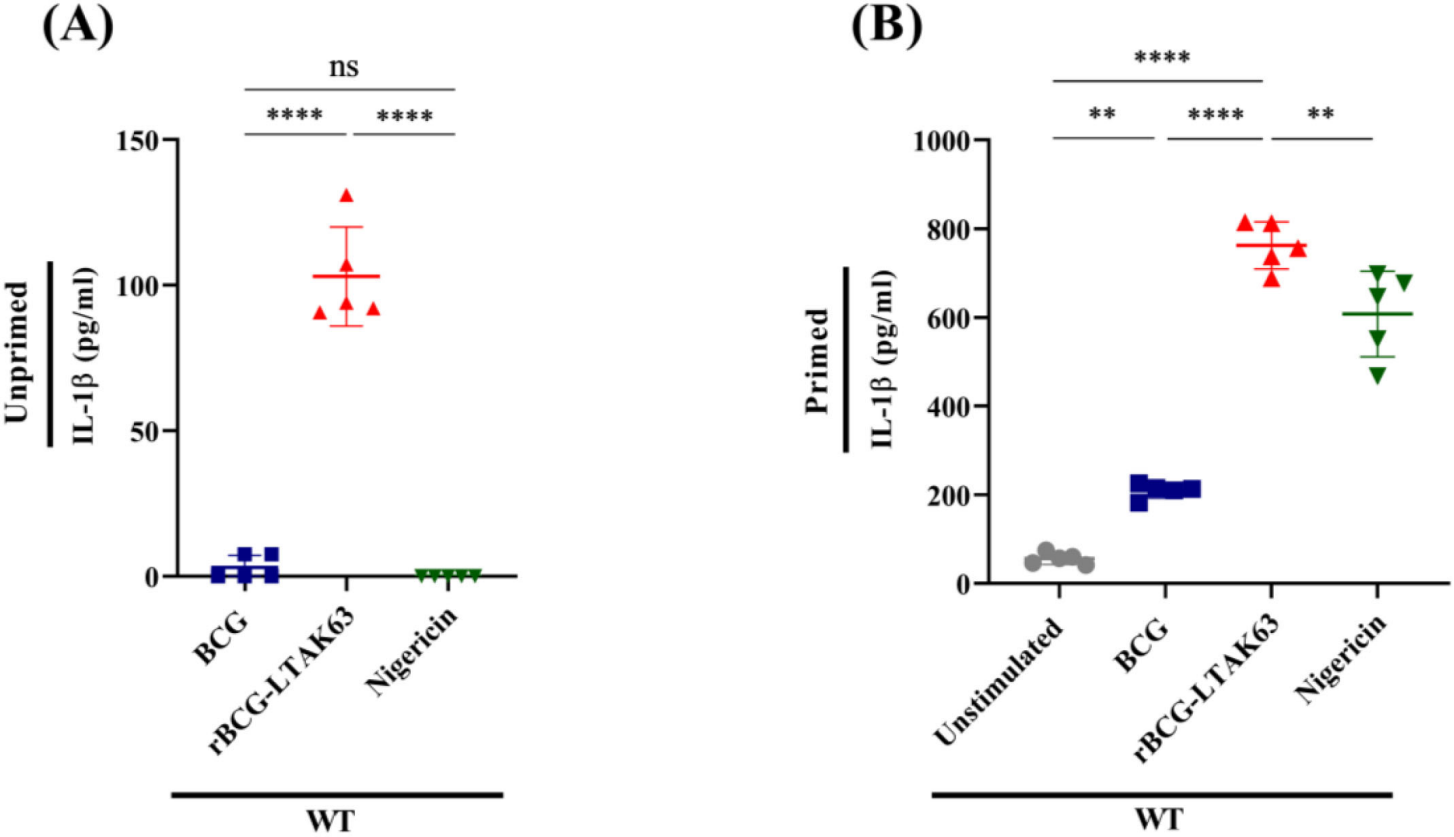
rBCG-LTAK63 induces BMDMs IL-1β production even without priming. BMDMs from C57Bl/6 mice were either primed with LPS (500 ng/mL, 4 h) **(A)** or not **(B)** and subsequently stimulated with culture medium alone (Unstimulated), BCG, or rBCG-LTAK63 (MOI 10:1) for 6 h. Nigericin (20 mM, 40 min), a potent NLRP3 agonist, served as a positive control. IL-1β levels were quantified in culture supernatants by ELISA. Data represent the mean ± SD of 5 replicates per group. Statistical significance was determined by one-way ANOVA and Tukey’s post hoc test (**p < 0.01, ****p < 0.0001, ns = not significant).

To evaluate inflammasome activation by rBCG-LTAK63, we measured IL-1β concentration in the supernatants of BMDMs according to standard protocols (23). Our results demonstrate that only rBCG-LTAK63 was able to induce detectable levels of IL-1β in the supernatant of non-primed cells (**Figure 3A**). On the other hand, when the BMDMs were primed with LPS, BCG induced IL-1β expression as compared to unstimulated cells, while rBCG-LTAK63 induced even higher levels (**Figure 3B**).

### 3.3 rBCG-LTAK63 induces IL-1β secretion through inflammasome-associated pathways

**Figure 4:**
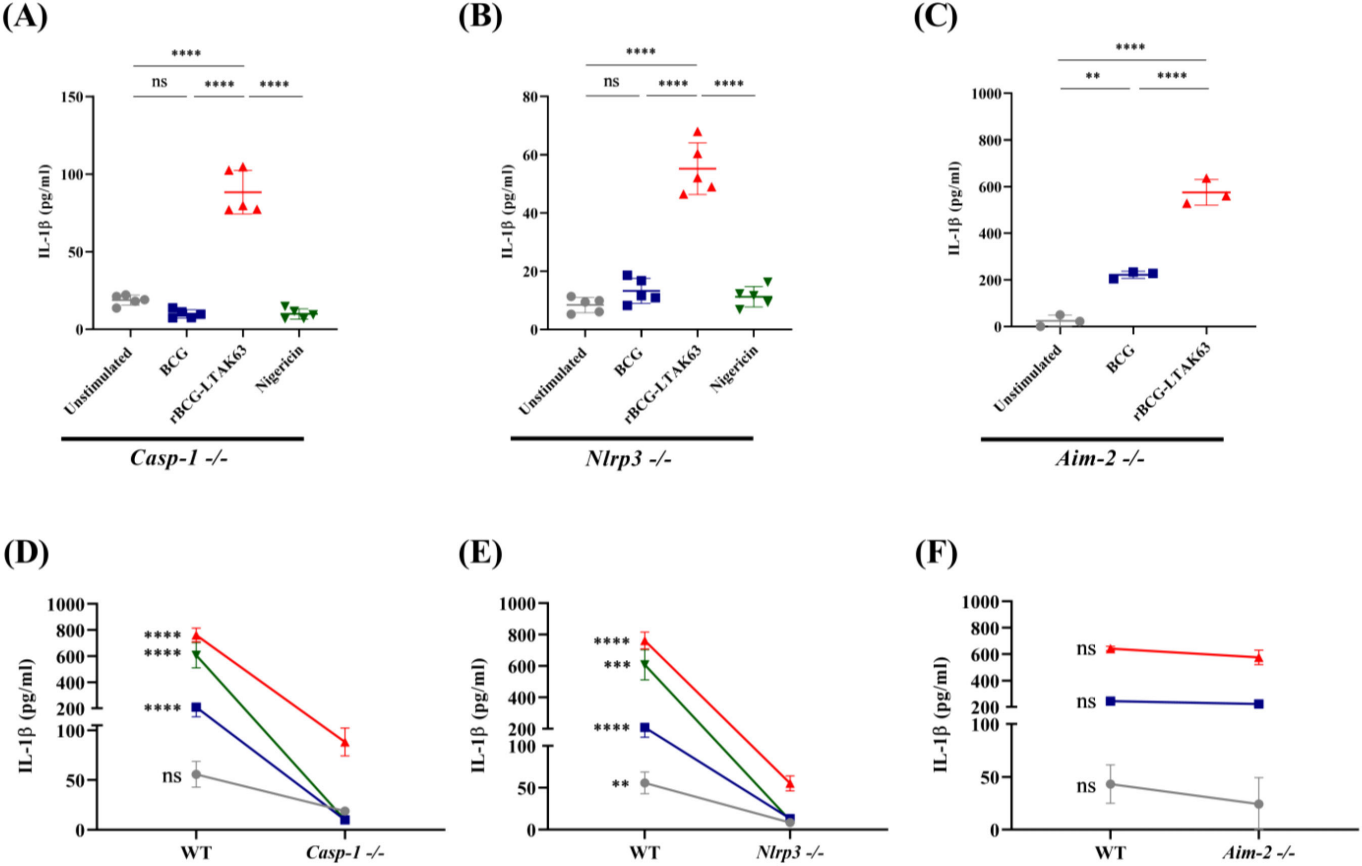
rBCG-LTAK63 induces IL-1β secretion via inflammasome-associated pathways. BMDMs from *Casp-1*-/-**(A)**, *Nlrp3*-/-**(B)**, and *Aim-2*-/- **(C)** mice were primed with LPS (500 ng/mL, 4 h) and subsequently stimulated with culture medium alone (Unstimulated), BCG, or rBCG-LTAK63 (MOI 10:1) for 6 (h) Nigericin (20 μM, 40 min) served as a positive control for Caspase-1 and NLRP3 activation. IL-1β levels were quantified in culture supernatants by ELISA. Comparison of IL-1β secretion in wild-type (WT) versus *Casp-1*-/-**(D)**, *Nlrp3*-/- **(E)** or *Aim-2*-/- **(F)**. Data represents the mean ± SD of 5 replicates per group. Statistical significance was determined by one-way ANOVA with Tukey’s *post hoc* test (**p < 0.01, ***p < 0.001, ****p < 0.0001, ns = not significant).

In order to identify the inflammasome pathway activated by rBCG-LTAK63 we used inflammasome-deficient macrophages. In *Casp-1*-/- or *Nlrp3*-/- macrophages, neither BCG nor the positive control nigericin could induce IL-1β secretion. In contrast, rBCG-LTAK63 stimulation of either *Casp-1*-/- or *Nlrp3*-/- macrophages induced IL-1β (**Figures 4A, B**). The production of IL-1β in *Aim-2*-deficient macrophages was maintained by both stimulus (**Figure 4C**). Comparative analyses across genotypes (e.g., wild-type vs. knockout) showed that *Casp-1*-/- or *Nlrp3*-/- cells exhibited an ~10-fold reduction in IL-1β compared to wild-type cells, whereas IL-1β production in *Aim-2*-/- macrophages was comparable to wild-type levels (**Figures 4D–F**).

Together, these results indicate that rBCG-LTAK63 induces IL-1β secretion predominantly through the canonical NLRP3/caspase-1 inflammasome pathway, while also engaging caspase-1 and NLRP3-independent mechanisms that partially compensate for the absence of these components.

### 3.4 Crosstalk between inflammasome-activated macrophages and T cells

To investigate the crosstalk between innate and adaptive immunity, we established a co-culture system combining inflammasome-activated BMDMs with naïve splenocytes or splenocytes from immunized mice (**Figure 2B**).

**Figure 5:**
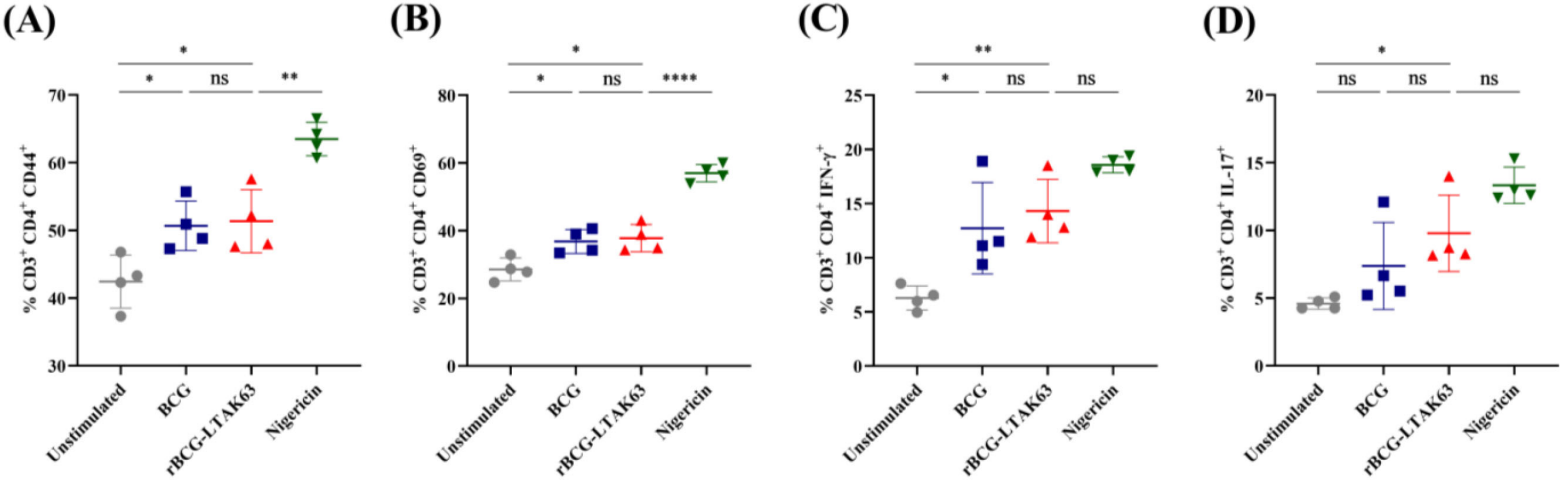
rBCG-LTAK63 enhances CD4^+^ T cell activation and polarization. Splenocytes from naïve C57Bl/6 mice were co-cultured with LPS-primed BMDMs previously stimulated with BCG or rBCG-LTAK63 (MOI 10:1, 6h) and T cell responses evaluated using flow cytometry **(A-D)**. **(A)** memory phenotype (CD44^+^), **(B)** early activation (CD69^+^), **(C)** IFN-γ^+^, and **(D)** IL-17^+^ populations. Data represents the mean ± SD of 4 replicates per group. Statistical significance was determined by one-way ANOVA with Tukey’s *post hoc* test (*p < 0.05, **p < 0.01, ***p < 0.001, ****p < 0.0001; ns = not significant).

When co-cultured with naïve splenocytes, macrophages stimulated with either BCG or rBCG-LTAK63 increased the percentage of memory-phenotype (CD44^+^, **Figure 5A**) and activated (CD69^+^, **Figure 5B**) CD4^+^ T cells, and promoted direction toward a Th1 phenotype (IFN-γ^+^, **Figure 5C**). However, no significant differences were observed between the BCG and rBCG-LTAK63 groups in these subsets, including Th17 (IL-17^+^) cells (**Figure 5D**).

In contrast, when the co-culture was performed using splenocytes from immunized mice (**Figure 2B**) a more pronounced effect was observed. rBCG-LTAK63-stimulated macrophages induced a significantly higher percentage of both activated (CD69^+^) and memory-phenotype (CD44^+^) CD4^+^ T cells compared to its own basal control (**Supplementary Figures S2D, E**). Under these conditions, rBCG-LTAK63 stimulation also led to a robust increase in IFN-γ^+^ (Th1) and IL-17^+^ (Th17) CD4^+^ T cells relative to its basal group (**Supplementary Figures S2F, G**). These findings demonstrate that pre-existing immunity unleashes rBCG-LTAK63’s unique capacity to drive a robust and polyfunctional adaptive immune response.

### 3.5 Innate immune competence is required for the enhanced immunogenicity of rBCG-LTAK63

We investigated the specific involvement of innate inflammatory responses using macrophages (BMDMs) from AIRmax and AIRmin mouse strains. These strains were genetically selected for high and low acute inflammatory responses, respectively. The AIRmin^TT^ strain is a subline with a characterized loss-of-function mutation in ASC (24).

**Figure 6:**
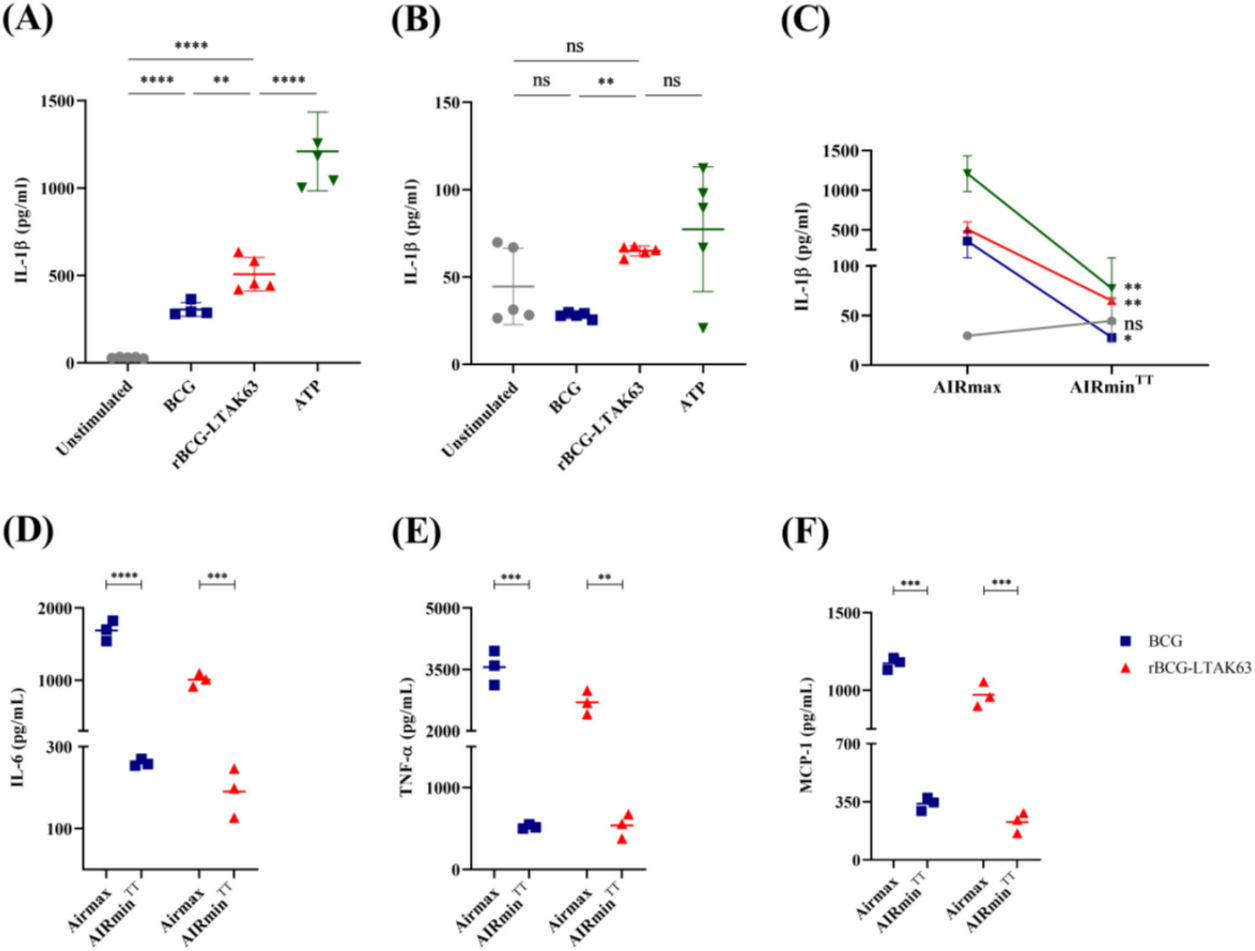
Host inflammatory response determines the cytokine secretion in rBCG-LTAK63-infected BMDMs. Bone marrow-derived macrophages (BMDMs) from AIRmax (control) and AIRmin^TT^ mice were primed with LPS (500 ng/mL, 4 h) and subsequently stimulated with medium (Unstimulated), BCG or rBCG-LTAK63 (MOI 10:1) for 6 (h) Cytokine production was measured in the supernatants. **(A, B)** IL-1β secretion in BMDMs from **(A)** AIRmax and **(B)** AIRmin^TT^ mice. ATP (5 mM, 30 min) was used as a positive control. Comparison of **(C)** IL-1β, **(D)** IL-6, **(E)** TNF-α, and **(F)** MCP-1 secretion between AIRmax and AIRmin^TT^ groups under each stimulus condition. Bars represent the mean ± SD of 4–5 replicates per group. Statistical significance was determined by one-way ANOVA with Tukey’s *post hoc* test (**p < 0.01, ***p < 0.001, ****p < 0.0001; ns = not significant).

In AIRmax macrophages, rBCG-LTAK63 triggered significantly higher IL-1β secretion than BCG or unstimulated cells (**Figure 6A**). In AIRmin^TT^ macrophages, the IL-1β response was completely abolished for all stimuli including the positive control ATP (**Figures 6B, C**). rBCG-LTAK63 was statistically different from BCG group but not in comparison to unstimulated cells (**Figure 6B**).

We also investigated whether rBCG-LTAK63 would also stimulate a cytokine response beyond IL-1β. The results showed a significant reduction in the production of IL-6 (**Figure 6D**), TNF-α (**Figure 6E**), and MCP-1 (**Figure 6F**) was observed in AIRmin^TT^ in comparison to AIRmax macrophages. Collectively, these data demonstrate that rBCG-LTAK63 can induce IL-1β secretion in both macrophage lineages but with a reduced efficiency in AIRmin BMDMs.

### 3.6 Protective effect of rBCG-LTAK63 immunization in AIRmax and AIRmin^TT^ mice

**Figure 7:**
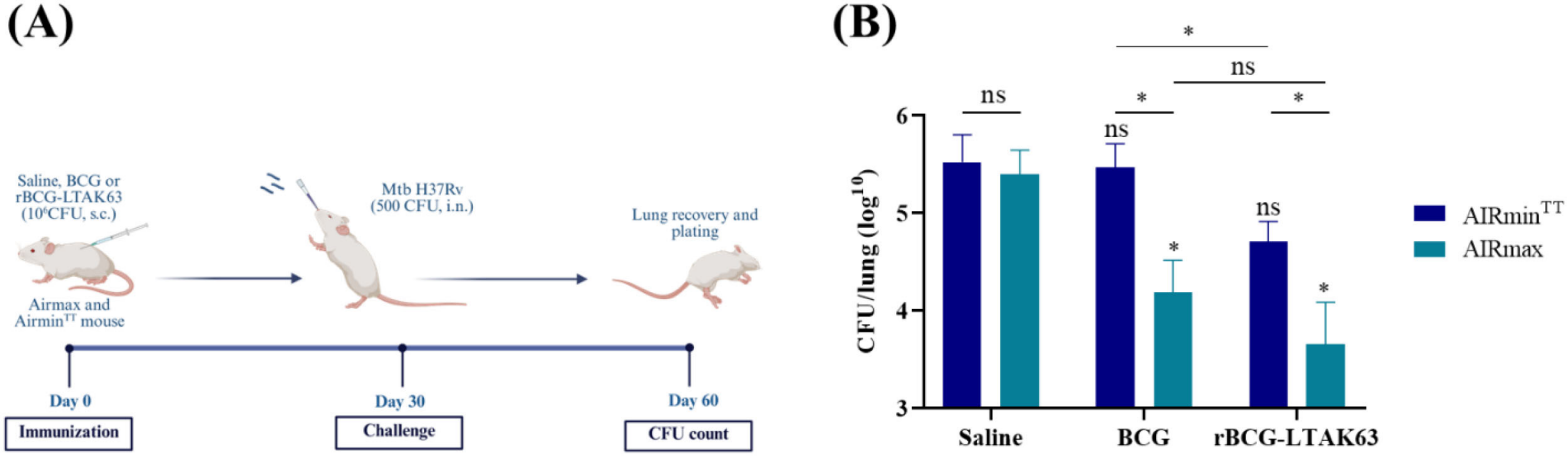
Protection against Mtb challenge induced by BCG or rBCG-LTAK63 in AIRmax and AIRmin^TT^ mice. **(A)** AIRmax and AIRmin^TT^ mice were divided into three groups (n = 5 mice per group) and received 100 µL saline, or immunized subcutaneously (s.c.) with BCG (10^6^ CFU) or rBCG-LTAK63 (10^6^ CFU). Thirty days post-immunization, animals were challenged intranasally with 500 CFU of *M. tuberculosis* H37Rv. At day 30 post-challenge, mice were euthanized, lungs were collected, and **(B)** bacterial burden was assessed by CFU counts. Statistical significance was analyzed using unpaired two-tailed Student’s *t*-tests for pairwise comparisons (*p* < 0.05; ns, not significant). Asterisks above bars indicate comparisons with the respective saline group.

rBCG-LTAK63 has consistently demonstrated increased protection of mice against Mtb challenge including in different BCG background strains (Moreau or Danish), expression systems (auxotrophic complementation or antibiotic-maintained), challenge models (intratracheal or intranasal) and Mtb strains (H37Rv or Beijing) (14, 15). Here, we used AIRmax and AIRmin mouse models to assess whether inflammasome-associated innate immune competence influences vaccine-induced protection *in vivo*. AIRmax and AIRmin^TT^ mice were immunized with either BCG or rBCG-LTAK63, or received saline, and subsequently challenged with Mtb H37Rv. The pulmonary bacterial burden was quantified 30 days post-infection (**Figure 7A**).

Non-immunized mice (Saline group) from both AIRmax and AIRmin^TT^ genotypes exhibited a similarly high bacterial load in the lungs of challenged mice (**Figure 7B**). In AIRmin^TT^ mice, BCG did not induce protection, but in AIRmax mice, protection was similar to that obtained in naïve mice. More importantly, rBCG-LTAK63 induced higher protection as compared to BCG in both genotypes. Comparison between genotypes demonstrates that AIRmax mice exhibit significantly lower CFU counts in comparison to AIRmin^TT^ mice in both BCG and rBCG-LTAK63 groups. Nevertheless, the ability of rBCG-LTAK63 to confer protection in AIRminTT mice indicates that this recombinant vaccine can partially overcome limitations imposed by a genetically attenuated inflammatory background.

## 4 Discussion

Inflammasomes play a critical role in innate immunity and the subsequent developed adaptive responses. Significant advances have been made in understanding inflammasome biology in the context of vaccine-induced immune responses. Inflammasome activation is not only important in mycobacterial infections but also contributes to the protective effect elicited by BCG, both as a vaccine and as immunotherapy in bladder cancer (25–27).

From the draining lymph nodes of immunized mice clear differences between rBCG-LTAK63 and the parental BCG were noted as early as 7 days post-immunization. The overrepresentation of a chaperone-centered hub is a response to a highly active and stressed cellular environment. In conjunction with the upregulation of the chemokines CXCL1, CXCL2 and COX-2 (Ptgs2), this signature implies an early and robust innate immune response induced by rBCG-LTAK63. The absence of a direct IL-1-related hub may be due to the predominance of T and B lymphocytes in the lymph nodes at 7 days post-immunization (28).

Our study demonstrates that rBCG-LTAK63 induces an inflammasome-associated innate immune response in a distinct manner compared to the parental BCG, characterized by the enhanced IL-1β secretion linked to the adjuvant LTAK63. IL-1β is observed even without LPS priming and suggests that rBCG-LTAK63 may simultaneously act as signal 1 (priming) and signal 2 (activation). While canonical NLRP3 signaling contributes to this response, the residual IL-1β production detected in *caspase-1*-/- and *Nlrp3*-/- macrophages indicates that additional pathways are participating. These may include caspase-8–dependent mechanisms can be recruited after the activation of certain surface receptors such as TLR-2 and Dectin-1. These mechanisms have not been associated with LT or LTA, but they have been described in the context of mycobacterial infection (29–32).

It also supports the evidence that even for BCG, a known immunostimulant, which comprises a milieu of multiple PAMPs that activate TLR, NLR, CLR and RLR downstream signaling (33), further reinforcement of innate immune pathways can be beneficial. The combination of BCG and a RIG-I/NOD2-activating molecule (Inarigivir) increased the secretion of pro-inflammatory cytokines, including IL-1β, and resulted in improved protection against Mtb (34). Similarly, VPM1002 (BCGΔureC::hly), which provides improved protection against Mtb, also triggers the AIM-2 inflammasome and increases IL-1β in comparison to parental BCG (8). In fact, combinatorial inflammasome activation using multiple ligands can trigger mechanisms not accessed by a single ligand (35). Therefore, co-activation of multiple innate pathways including inflammasomes may result in improved immune outcomes.

The interplay between inflammasome activation and adaptive immunity was also evaluated. Overall, rBCG-LTAK63-stimulated macrophages drove a significant CD4^+^ T cell activation and Th1/Th17 polarization in co-culture experiments. These observations extend previous reports linking IL-1β to Th17 differentiation (36). Furthermore, a robust innate response is not only important to promote proper adaptive responses but may directly provide innate-mediated protection independent of T cells (37).

Genetic models revealed that IL-1β secretion was dependent on the mouse phenotype, with AIRmax mice mounting stronger inflammatory responses than AIRmin (24). Accordingly, not only IL-1β but other inflammatory cytokines/chemokines are upregulated in AIRmax mice upon stimulation (e.g. Cxcl1, Cxcl2, Tnf-α, Il-6, Ccl2) (38). The naturally occurring Pycard missense mutation (E19K) present in AIRmin^TT^ mice impairs, but does not abolish, ASC function, resulting in an attenuated inflammasome-associated response influenced by genetic background (24). Together, these characteristics make AIRmax and AIRmin^TT^ strains valuable models for studying the relationship between innate inflammation and immune-mediated outcomes (21, 24). Interestingly, when challenged with Mtb, rBCG-LTAK63 induced protection in both genotypes, whereas BCG was only effective in AIRmax mice. Even if other mechanisms are present aside differences in inflammatory responses, these results suggests that rBCG-LTAK63 can bypass certain genetic restraints to induce protection. Engagement of inflammasome-associated innate pathways correlates with a reduced pulmonary bacterial burden in both BCG and rBCG-LTAK63 immunized mice, suggesting a contributory, rather than exclusive, role for inflammasome signaling in protection. How rBCG-LTAK63 induces protection in AIRmin^TT^ mice despite their impaired innate inflammatory response remains to be evaluated. The renewed interest in the innate responses induced by BCG or its recombinant derivatives seems to point towards common pathways such as cGAS/STING (39) or autophagy (40). Autophagy can be induced in situations of cellular stress, and the transcriptional profile of rBCG-LTAK63-immunized mice suggests an intense cellular stress. In fact, a recent work describes the induction of autophagy by rBCG-LTAK63 in RAW264.7, a murine monocyte/macrophage-like cell line (41). Additionally, other T cell subsets (memory-like or effector function) can be generated independent of inflammasomes or other innate mechanisms and may be involved in the induced protection (17).

Overall, our findings demonstrate that rBCG-LTAK63 enhances innate immune activation, including inflammasome-associated pathways, and that this heightened innate response correlates with improved protection against *M. tuberculosis*. Rather than establishing a direct causal dependence on NLRP3 or ASC, our data support a model in which broad and effective activation of innate immune mechanisms contributes to the superior efficacy of rBCG-LTAK63.

## 5 Conclusion

Our findings demonstrate that the rBCG-LTAK63 vaccine promotes robust innate immune activation, including inflammasome-associated pathways, which likely contributes to its enhanced protective efficacy against *M. tuberculosis*. Future studies will be required to define the precise contribution of individual inflammasome components and to investigate the involvement of other innate immune mechanisms. This work highlights the importance of innate immune activation as a central axis in tuberculosis vaccine development and supports the continued evaluation of rBCG-LTAK63 as a promising next-generation tuberculosis vaccine.

## Data Availability

The RNA-seq data are available in the NCBI Sequence Read Archive under accession number GSE278523.
