# Supplementary material for: Broad innate immune activation enhances the protective efficacy of rBCG-LTAK63 against Mycobacterium tuberculosis
Source: Front Immunol. 2026 Mar 4;17:1758476. doi: 10.3389/fimmu.2026.1758476 (PMC12996045; doi:10.3389/fimmu.2026.1758476)
Supplement: Supplementary file 1 [file DataSheet1.docx]

Supplementary Figures

Broad Innate Immune Activation Enhances the Protective Efficacy of rBCG-LTAK63 Against Mycobacterium tuberculosis

Ana Carolina de Oliveira Carvalho^1^, Monalisa Martins Trentini^1^, Dunia Rodriguez^1^, Lázaro Moreira Marques-Neto^1^, Paulo Henrique Santana Silveira^1^, Nancy Starobinas^2^, Sergio Costa Oliveira^3^, Luciana Cezar de Cerqueira Leite^1^ and Alex Issamu Kanno^1*^

^1^ Laboratório de Desenvolvimento de Vacinas, Instituto Butantan, São Paulo, Brazil

^2^ Laboratório de Imunogenética, Instituto Butantan, São Paulo, Brazil

^3^ Departamento de Imunologia, Instituto de Ciências Biomédicas, Universidade de São Paulo, São Paulo, Brazil

***** Alex Issamu Kanno
Email: [alex.kanno@butantan.gov.br](mailto:alex.kanno@butantan.gov.br)

Postal address: Laboratório de Desenvolvimento de Vacinas, Instituto Butantan, Avenida Doutor Vital Brasil, 1500, 05503-900, São Paulo, SP, Brazil.


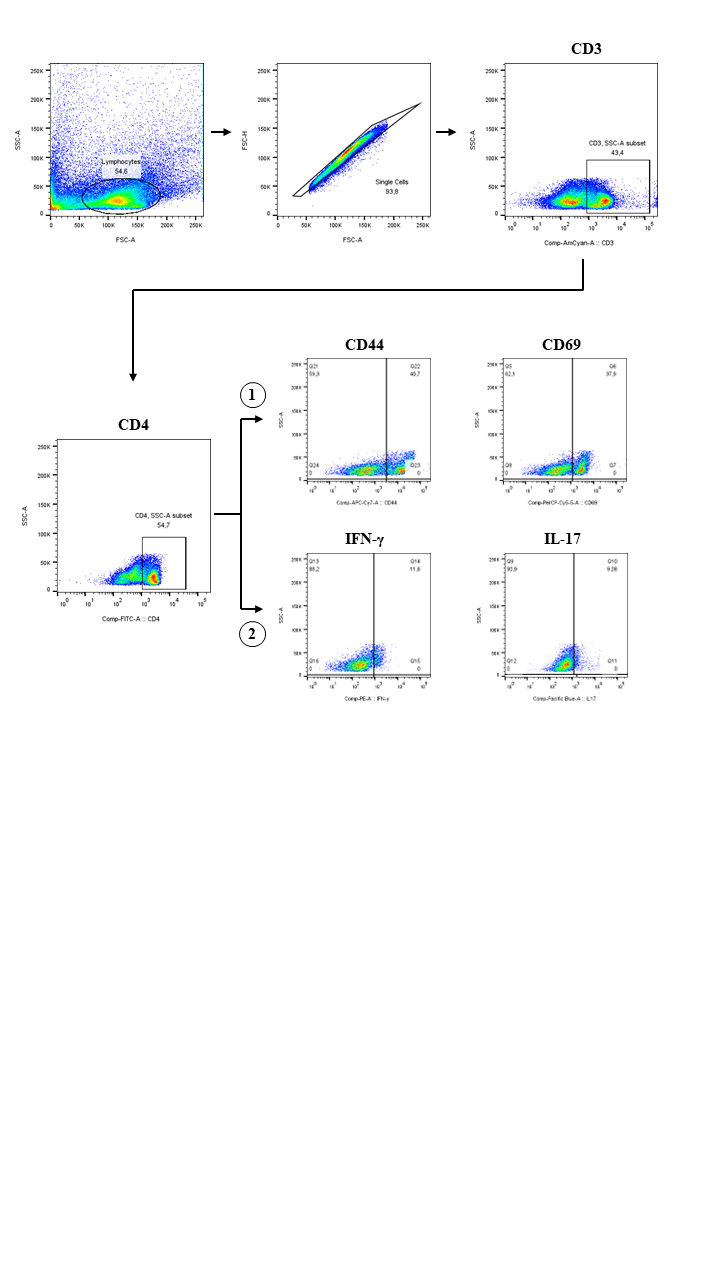


**Supplementary Figure S1:** **Gating strategy.** Lymphocytes were selected based on size (FSC) and granularity (SSC) within singlet events. **(1)** CD4⁺ T cells were gated to determine the frequency of CD44⁺ and CD69⁺ cells. **(2)** CD4⁺ T cells were also analyzed for intracellular cytokine expression to quantify IFN-γ⁺ and IL-17⁺ populations.

| **#term ID** | **term description** | **observed gene count** | **background gene count** | **strength** | **signal** | **false discovery rate** | **matching proteins in your network (labels)** |
| --- | --- | --- | --- | --- | --- | --- | --- |
| **mmu04141** | Protein processing in endoplasmic reticulum | 9 | 169 | 1.26 | 1.51 | 7.86E-07 | Dnajb1,Hspa8,Hspa1a,Hsp90aa1,Atf4,Ppp1r15a,Dnaja1,Hspa1b,Hsph1 |
| **mmu04657** | IL-17 signaling pathway | 7 | 89 | 1.43 | 1.56 | 2.09E-06 | Fosb,Fos,Cxcl1,Ptgs2,Cxcl2,Hsp90aa1,Jun |
| **mmu04668** | TNF signaling pathway | 7 | 112 | 1.33 | 1.39 | 6.22E-06 | Fos,Cxcl1,Ptgs2,Junb,Cxcl2,Jun,Atf4 |
| **mmu04010** | MAPK signaling pathway | 9 | 287 | 1.03 | 1.11 | 1.29E-05 | Gadd45b,Hspa8,Fos,Dusp1,Hspa1a,Jun,Atf4,Hspa1b,Nr4a1 |
| **mmu04915** | Estrogen signaling pathway | 7 | 131 | 1.26 | 1.28 | 1.29E-05 | Hspa8,Fos,Hspa1a,Hsp90aa1,Jun,Atf4,Hspa1b |
| **mmu05145** | Toxoplasmosis | 6 | 106 | 1.29 | 1.17 | 5.09E-05 | Hspa8,Igtp,Irgm1,Irgm2,Hspa1a,Hspa1b |
| **mmu05134** | Legionellosis | 5 | 60 | 1.45 | 1.22 | 6.22E-05 | Hspa8,Cxcl1,Cxcl2,Hspa1a,Hspa1b |
| **mmu04621** | NOD-like receptor signaling pathway | 7 | 193 | 1.09 | 1.02 | 7.91E-05 | Gbp2b,Cxcl1,Gbp7,Cxcl2,Hsp90aa1,Gbp3,Jun |
| **mmu05031** | Amphetamine addiction | 4 | 63 | 1.34 | 0.83 | 0.0016 | Fosb,Fos,Jun,Atf4 |
| **mmu04210** | Apoptosis | 5 | 135 | 1.1 | 0.74 | 0.0019 | Gadd45b,Fos,Pmaip1,Jun,Atf4 |
| **mmu05162** | Measles | 5 | 140 | 1.09 | 0.73 | 0.002 | Hspa8,Fos,Hspa1a,Jun,Hspa1b |
| **mmu04612** | Antigen processing and presentation | 4 | 75 | 1.26 | 0.77 | 0.0023 | Hspa8,Hspa1a,Hsp90aa1,Hspa1b |
| **mmu05210** | Colorectal cancer | 4 | 86 | 1.2 | 0.72 | 0.0033 | Gadd45b,Fos,Pmaip1,Jun |
| **mmu05323** | Rheumatoid arthritis | 4 | 84 | 1.21 | 0.72 | 0.0033 | Fos,Cxcl1,Cxcl2,Jun |
| **mmu04064** | NF-kappa B signaling pathway | 4 | 99 | 1.14 | 0.65 | 0.0052 | Gadd45b,Cxcl1,Ptgs2,Cxcl2 |
| **mmu04380** | Osteoclast differentiation | 4 | 119 | 1.06 | 0.58 | 0.0086 | Fosb,Fos,Junb,Jun |
| **mmu05030** | Cocaine addiction | 3 | 47 | 1.34 | 0.63 | 0.0086 | Fosb,Jun,Atf4 |
| **mmu05167** | Kaposi sarcoma-associated herpesvirus infection | 5 | 210 | 0.91 | 0.55 | 0.0086 | Fos,Cxcl1,Ptgs2,Cxcl2,Jun |
| **mmu04213** | Longevity regulating pathway - multiple species | 3 | 62 | 1.22 | 0.55 | 0.0154 | Hspa8,Hspa1a,Hspa1b |
| **mmu05200** | Pathways in cancer | 7 | 527 | 0.66 | 0.43 | 0.0154 | Gadd45b,Fos,Pmaip1,Ptgs2,Cxcr4,Hsp90aa1,Jun |
| **mmu05418** | Fluid shear stress and atherosclerosis | 4 | 142 | 0.98 | 0.51 | 0.0154 | Fos,Dusp1,Hsp90aa1,Jun |
| **mmu05020** | Prion disease | 5 | 263 | 0.81 | 0.46 | 0.0164 | Hspa8,Stip1,Hspa1a,Atf4,Hspa1b |
| **mmu05140** | Leishmaniasis | 3 | 66 | 1.19 | 0.53 | 0.0164 | Fos,Ptgs2,Jun |
| **mmu04061** | Viral protein interaction with cytokine and cytokine receptor | 3 | 87 | 1.07 | 0.44 | 0.0323 | Cxcl1,Cxcr4,Cxcl2 |
| **mmu04659** | Th17 cell differentiation | 3 | 101 | 1.01 | 0.39 | 0.0466 | Fos,Hsp90aa1,Jun |

**Supplementary Table S1. KEGG pathway over-representation analysis (ORA) results for differentially expressed genes (DEGs) comparing rBCG-LTAK63 vs. BCG in the draining lymph nodes of vaccinated mice at day 7 post-immunization.** Each row reports the KEGG pathway ID and description, the observed gene count (DEGs mapped to the pathway), the background gene count (genes annotated to the pathway in the reference), enrichment metrics (strength and signal), and the false discovery rate (FDR). The last column lists the matching proteins in the user’s network with the corresponding gene labels contributing to each enriched pathway.


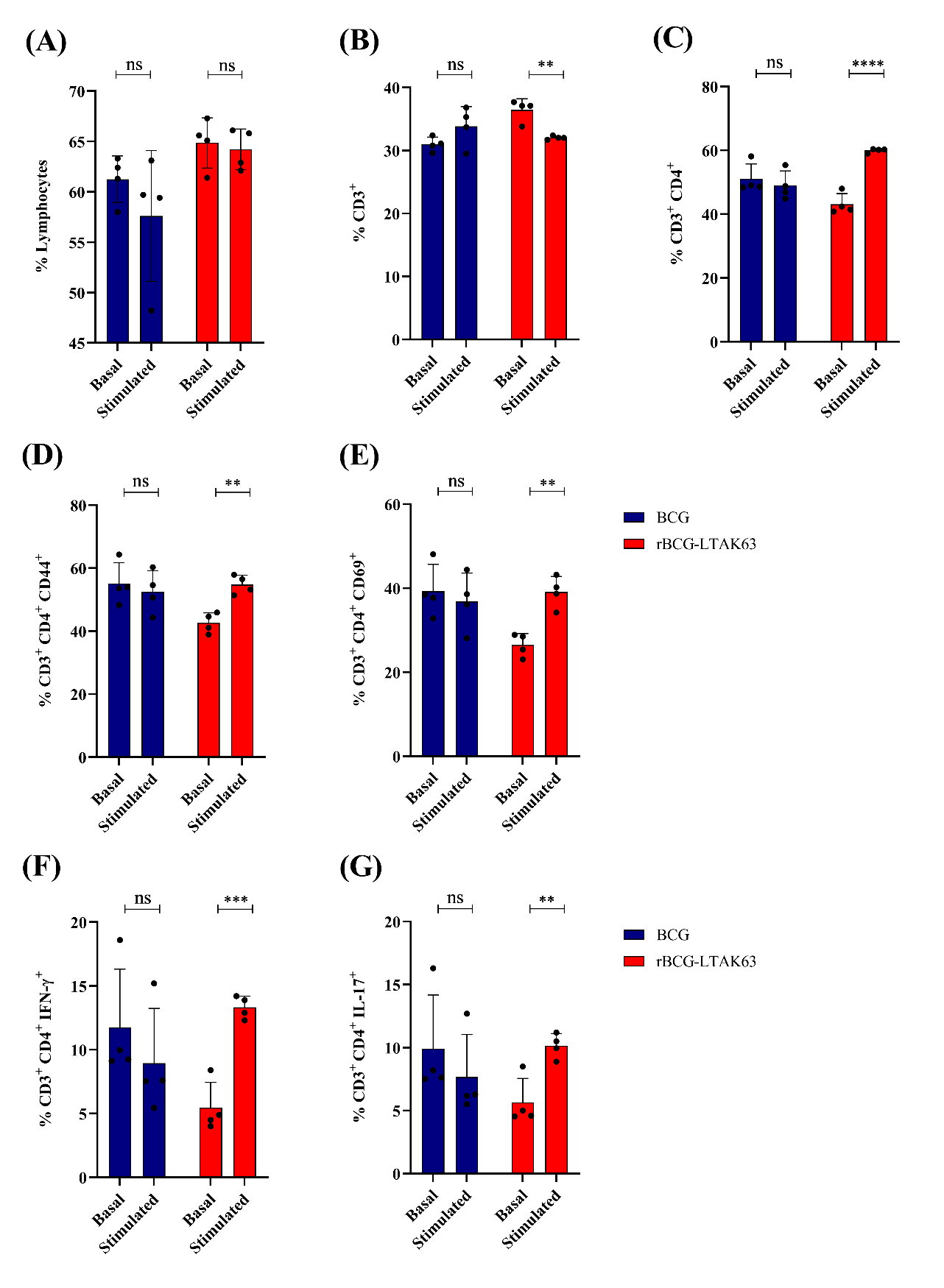


**Supplementary Figure S2: rBCG-LTAK63 enhances T lymphocyte activation in macrophage-splenocyte co-cultures.** Splenocytes from C57Bl/6 mice immunized with 10⁶ CFU of BCG or rBCG-LTAK63 were co-cultured with LPS-primed bone marrow-derived macrophages (BMDMs) that had been previously infected with the respective strains (MOI 10:1, 6h). T cell responses were evaluated by flow cytometry. Blue plots represent co-cultures where both BMDMs and splenocytes were from BCG-treated groups. Red plots represent co-cultures where both BMDMs and splenocytes were from rBCG-LTAK63-treated groups. Analysis of **(A)** total lymphocytes, **(B)** CD3⁺ T cells, **(C)** CD3⁺CD4⁺ T helper cells, **(D)** memory phenotype (CD44⁺), **(E)** early activation (CD69⁺), **(F)** IFN-γ⁺, and **(G)** IL-17⁺ populations. Data are presented as mean ± SD (n=4). Statistical significance was determined by one-way ANOVA with Tukey's post hoc test (*p < 0.05, **p < 0.01, ***p < 0.001, ****p < 0.0001; ns, not significant).
